# Improvement of the performance of survival prediction in the ageing blunt trauma population: A cohort study

**DOI:** 10.1371/journal.pone.0209099

**Published:** 2018-12-18

**Authors:** Leonie de Munter, Nancy C. W. ter Bogt, Suzanne Polinder, Charlie A. Sewalt, Ewout W. Steyerberg, Mariska A. C. de Jongh

**Affiliations:** 1 Department Trauma TopCare, Elisabeth-TweeSteden Hospital (ETZ Ziekenhuis), Tilburg, the Netherlands; 2 Network Emergency Care Euregio, Enschede, the Netherlands; 3 Department of Public Health, Erasmus Medical Centre, Rotterdam, the Netherlands; 4 Department of Biomedical Data Sciences, Leiden University Medical Center, Leiden, The Netherlands; 5 Brabant Trauma Registry, Network Emergency Care Brabant, Tilburg, the Netherlands; Monash University School of Public Health and Preventive Medicine, AUSTRALIA

## Abstract

**Introduction:**

The overestimation of survival predictions in the ageing trauma population results in negative benchmark numbers in hospitals that mainly treat elderly patients. The aim of this study was to develop and validate a modified Trauma and Injury Severity Score (TRISS) for accurate survival prediction in the ageing blunt trauma population.

**Methods:**

This retrospective study was conducted with data from two Dutch Trauma regions. Missing values were imputed. New prediction models were created in the development set, including age (continuous or categorical) and Anesthesiologists Physical Status (ASA). The models were externally validated. Subsets were created based on age (≥75 years) and the presence of hip fracture. Model performance was assessed by proportion explained variance (Nagelkerke R^2^), discrimination (Area Under the curve of the Receiver Operating Characteristic, AUROC) and visually with calibration plots. A final model was created based on both datasets.

**Results:**

No differences were found between the baseline characteristics of the development dataset (n = 15,530) and the validation set (n = 15,504). The inclusion of ASA in the prediction models showed significant improved discriminative abilities in the two subsets (e.g. AUROC of 0.52 [95% CI: 0.46, 0.58] vs. 0.74 [95% CI: 0.69, 0.78] for elderly patients with hip fracture) and an increase in the proportion explained variance (R^2^ = 0.32 to R^2^ = 0.35 in the total cohort). The final model showed high agreement between observed and predicted survival in the calibration plot, also in the subsets.

**Conclusions:**

Including ASA and age (continuous) in survival prediction is a simple adjustment of the TRISS methodology to improve survival predictions in the ageing blunt trauma population. A new model is presented, through which even patients with isolated hip fractures could be included in the evaluation of trauma care.

## Introduction

Accurate survival predictions are necessary for reliable comparisons of the quality of care between centers. The Dutch Trauma Registry (DTR) is a nationwide registry collecting trauma data of approximately 80.000 admitted patients annually in the Netherlands[1,2]. The DTR updated the coefficients of the Trauma and Injury Severity Score (TRISS) and used this updated TRISS for evaluation of trauma care[1,3]. This model has accurate survival predictions when looking at the trauma population in general, but showed an overestimation of survival in the elderly trauma patient[4,5].

Patients with isolated hip fractures are often excluded from trauma registries[6]. Nevertheless, the purpose of the trauma registry is to document and gain insight into the full spectrum of admitted trauma patients, including the elderly[7]. In 2016, 18.2% of the Dutch population was aged 65 years or older and it is expected that this number will increase to 26.5% in 2040[8]. Because the elderly remain more active later in life, it is likely that the proportion of elderly trauma patients will increase as well. Hence, the Dutch trauma registry includes patients with isolated hip fractures, and includes them for the evaluation of quality of care. Currently, almost 20% of the registry comprises elderly patients with hip fracture. Because survival predictions will be overestimated in the elderly, the benchmark numbers (e.g. W-statistic [Ws][9]) provided from the updated TRISS are negatively biased, especially in hospitals that mainly treat elderly patients[5].

Previously developed scoring systems for elderly with hip fracture, like the Nottingham Hip Fracture Score[10], are often based on variables that are not collected in the Dutch trauma registry (e.g. comorbidities present at time of hip fracture[11,12], the abbreviated mental test score [AMTS][13] or frailty[11,13]) and could therefore not be applied to the Dutch trauma population. Other previously developed models based on the TRISS methodology incorporated age as a categorical or continuous predictor and added comorbidity to the survival prediction model[14–19]. Although these models have the potential for accurate predictions in the total (and ageing) Dutch trauma population, the models were not solely assessed to the elderly trauma population and patients with isolated hip fractures were often excluded from the analyses.

Benchmark numbers should be comparable and accurate among all trauma subsets. Predictors for survival models should be reliable for the total trauma population and should be readily available from the trauma registry. The aim of this study was to develop and validate a modified TRISS with simple and minimal adjustments with variables available in the Dutch trauma registry.

## Methods

### Patient selection

This research was a retrospective cohort, conducted with registry data from two of the eleven trauma regions in the Netherlands: Network Emergency Care Brabant and Network Emergency Care Euregio. The first region included 12 emergency departments and was located in the South of the Netherlands, and the latter region was located in the east of the Netherlands with 4 emergency departments. Both regions included one level I trauma center and both regions included rural as urban areas.

The registry collected data from patients with injury that were admitted to one of the hospitals of the two regions after visiting the Emergency Department (ED) within 48 hours after trauma, independent of injury severity. Also, patients who died in the ED or secondary referrals were registered. Patients who were dead on arrival were excluded. Data was anonymized prior to access.

Two datasets were created, based on year of admission. The development set consisted of all observations from 2015 from the two regions (N = 16,095), including elderly patients (with hip fracture). The validation set consisted of all observations from 2016 (N = 16,073), including elderly patients (with hip fracture).

### Data collection and predictors

Information about the injury, prehospital and hospital physiological data, Abbreviated Injury Scale (2008) (AIS08)[20], and demographic variables were collected. The Dutch trauma registry did not include information about comorbidities other than the Anesthesiologists Physical Status (ASA)[21].

The prehospital Eye (E), Motor (M), and Verbal (V) components of the Glasgow Coma Scale (GCS)[22] and prehospital Respiratory Rate (RR) were used for patients who were sedated before arrival in the hospital. Also, the prehospital value for the V component of the GCS and RR were selected for intubated patients. Patterns of missing values for the survival predictors were analyzed. Missing values were considered Missing at Random (MAR) and missing predictor variables were imputed according to multiple imputation[23]. Missing values were imputed 30 times in both the development and validation set, according to the maximum percentage of missing values. The development set consisted of 3.5%, 3.6%, 3.7%, 28.8%, 9.9%, 1.1% and 9.2% missing values for E, M, V, RR, Systolic Blood Pressure (SBP), ISS and ASA respectively. The validation set consisted of 2.1%, 2.1%, 2.2%, 27.0%, 8.9%, 0.7% and 8.2% missing values for E, M, V, RR, SBP, ISS and ASA respectively. The imputation processes were assessed with convergence plots, which showed no trends.

Patients with penetrating injury (development set: N = 523 [3.2%] and validation set: N = 525 [3.3%]) were excluded, because the number of deaths was too low to assess the model performances adequately. Also, patients with unknown mechanism of injury (development set: N = 42 [0.3%] and validation set: N = 47 [0.3%]) were excluded from further analyses.

### Model development

Coefficients were calculated for five different models in the development dataset, with increasing number of parameters in the models and in-hospital mortality as outcome (Table 1). Model 1 is the updated TRISS as used in the Dutch Trauma Registry, with coefficients from 2015^1^. The other models were adjusted with age as categorical or continuous variable, and/or ASA was added to the model. The assumption of linearity in the logit was assessed for all linear variables.

**Table 1:** Variables that are incorporated in the different models.

	**GCS**	**SBP**	**RR**	**ISS**	**Age**			**ASA**
	Coded^a^	Coded^a^	Coded^a^	Linear	Dichotomous	Categorical	continuous	Categorical^b^
**Model 1**	X	X	X	X	X			
**Model 2**	X	X	X	X	X			X
**Model 3**	X	X	X	X		X		
**Model 4**	X	X	X	X		X		X
**Model 5**	X	X	X	X			X	
**Model 6**	X	X	X	X			X	X

Abbreviations: ASA, Anesthesiologists Physical Status; GCS, Glasgow Coma Scale; ISS, Injury Severity Score; RR, Respiratory Rate; SBP, Systolic Blood Pressure.

^**a**^Variables were coded according to the Revised Trauma Score calculations.

^b^ASA classification; ASA-1: a normal healthy patient, ASA-2: a patient with mild systemic disease, ASA-3: a patient with severe systemic disease, ASA-4: a patient with severe systemic disease that is a constant threat to life.

If no deviant model performances were found between the development dataset and the validation dataset because characteristics between sets were closely related, a final model was developed in a combined dataset (combining development dataset and validation dataset, N = 31,034)[24]. Year of admission was included and assessed as predictor in this final model.

### Subsets

The models were developed in the total trauma population. Because previous research showed poor performance of the updated TRISS in the elderly with and without hip fracture[5], two subsets were created in both the development and the validation dataset to validate the performance of the new models. The first subset consisted of elderly patients ≥75 years. The second subset consisted of patients suffering hip fracture, defined as ≥65 years with AIS08-codes 853161.3, 853162.3, 853151.3 and 853152.3, and ISS≤13.

### Statistical analysis

Data was reported according to the Transparent Reporting of a multivariable prediction model for individual Prognosis Or Diagnosis (TRIPOD) statement[25]. Because the models were pre-specified, the shrinkage principle is applied; the regression coefficients were meant for less extreme predictions, i.e. a better calibration. A shrinkage factor was calculated with s as uniform shrinkage factor and shrunk regression coefficients were calculated as s*β. The shrinkage factor (s) is based on the following formula:

s = (Model χ ^2^ –df) / Model χ^2^, with model χ^2^ as the difference in 2log likelihood between the model with and without predictors and *df* as the degrees of freedom of the number of predictors considered for the model[26,27]. The intercept was recalculated, based on the shrunken coefficients.

The proportion of variance that is explained by the model is calculated with Nagelkerke R-square (R^2^)[28]. Model performance was assessed by discrimination and calibration. Discrimination was measured using the Area Under the curve of the Receiver Operating Characteristic (AUROC). Differences between AUROC were considered significant when the 95% Confidence Intervals (CI) did not overlap, implying a p-value<0.01 for the difference in AUROC. Calibration was assessed visually with calibration plots. The models were externally validated by calculating the survival prediction for each model using the shrunken coefficients in the validation set, and were assessed on performance in both the validation set as in its subsets.

Data cleaning and multiple imputation were done using IBM SPSS version 24 (Chicago, USA). R version 3.4.0 (R Foundation for Statistical Computing, Vienna, Austria) was used for the drawing of the calibration curves. Calibration curves were created based on cubic splines.

## Results

### Patient characteristics

#### Development set

A total of 15,530 observations were used for the model development (Table 2). The mortality rate in the total population was 2.4% (n = 375) and 49.4% (n = 7,672) was male. Mean age was 54.8 years (SD: 29.1) and the median (Interquartile Range [IQR]) ISS was 4 (2–9). The population consisted of 5,369 patients equal to or older than 75 years and a total of 2,599 patients (16.7%) were ≥65 years with a hip fracture.

**Table 2:** Patient characteristics for the development and validation set.

		**Development set**			**Validation set**		
		Total	≥75 years	≥65 years with hip#^a^	Total	≥75 years	≥65 years with hip#^a^
**N**		15,530	5,369	2,599	15,504	5,405	2,689
**Age (mean, SD)**		54.8 (29.1)	84.2 (7.0)	81.8 (8.0)	54.8 (29.2)	84.1 (7.1)	81.8 (8.0)
**Male (N, %)**		7672 (49.4)	2572 (34.6)	801 (30.8)	7764 (50.1)	2584 (35.0)	774 (28.2)
**ASA (N, [%])^b^**							
	1	6865 (44.2)	403 (7.5)	229 (8.8)	6898 (44.5)	397 (7.3)	231 (8.6)
	2	5649 (36.4)	2773 (51.6)	1280 (49.2)	5630 (36.3)	2824 (52.2)	1301 (48.4)
	3	2928 (18.9)	2140 (39.9)	1062 (40.9)	2842 (18.3)	2106 (39.0)	112 (4.2)
	4	88 (0.6)	53 (1.0)	28 (1.1)	134 (0.9)	78 (1.4)	45 (1.7)
**Mortality (N, %)**		375 (2.4)	279 (5.2)	205 (4.2)	322 (2.1)	233 (4.3)	179 (3.8)
**E (N, [%])^c^**							
	Normal	14462 (93.1)	5035 (93.8)	2490 (95.8)	14626 (94.3)	5164 (95.5)	2604 (96.8)
	Abnormal	1068 (6.9)	334 (6.2)	109 (4.2)	878 (5.7)	241 (4.5)	85 (3.2)
**M (N, [%])^c^**							
	Normal	14675 (94.5)	5087 (94.7)	2490 (95.8)	14889 (96.0)	5209 (96.4)	2606 (96.9)
	Abnormal	855 (5.5)	282 (5.3)	109 (4.2)	615 (4.0)	196 (3.6)	83 (3.1)
**V (N, [%])^c^**							
	Normal	13971 (90.0)	4832 (90.0)	2398 (92.3)	14058 (90.7)	4903 (90.7)	2491 (92.6)
	Abnormal	1559 (10.0)	537 (10.0)	201 (7.7)	1446 (9.3)	502 (9.3)	198 (7.4)
**RR (N, [%])^c^**							
	Normal	15203 (97.9)	5267 (98.1)	2554 (98.3)	15148 (97.7)	5297 (98.0)	2649 (98.5)
	Abnormal	327 (2.1)	102 (1.9)	45 (1.7)	356 (2.3)	108 (2.0)	40 (1.5)
**SBP (N, [%])^c^**							
	Normal	14995 (96.6)	5262 (98.0)	2559 (40)	15050 (97.1)	5306 (98.2)	2659 (98.9)
	Abnormal	535 (3.4)	107 (2.0)	40 (1.5)	454 (2.9)	99 (1.8)	30 (1.1)
**ISS (median, IQR)**		4 (2, 9)	9 (4, 9)	9 (9, 9)	4 (2, 9)	9 (4, 9)	9 (9, 9)

Abbreviations: ASA, Anesthesiologists Physical Status; E, Eye component of the Glasgow Coma Scale; hip#, hip fracture; IQR, Inter Quartile Range; ISS, Injury Severity Score; M, Motor component of the Glasgow Coma Scale; ref, reference group; RR, Respiratory Rate; SBP, Systolic Blood Pressure; V, Verbal component of the Glasgow Coma Scale.

^a^Patients with hip fractures were defined as ≥65 years with AIS08-codes 853161.3, 853162.3, 853151.3 and 853152.3, and ISS≤13.

^b^ASA classification; ASA-1: a normal healthy patient, ASA-2: a patient with mild systemic disease, ASA-3: a patient with severe systemic disease, ASA-4: a patient with severe systemic disease that is a constant threat to life.

^c^Normal values for E, M and V were 4, 6 and 5 respectively. Normal value of RR was considered between 10 and 29 per minute and the normal value for SBP was >89 mm Hg.

### Validation set

A total of 15,504 observations were used for external validation (Table 2). The mortality rate in the validation set was 2.1% (n = 322) and 50.1% (n = 7,764) was male. Mean age was 54.8 years (SD: 29.2) and the median (Interquartile Range [IQR]) ISS was 4 (2–9). A total of 5,405 patients were equal to or older than 75 years and a total of 2,689 patients (17.3%) were ≥65 years with a hip fracture. No differences were found between the baseline characteristics of the development dataset and the validation set.

### Performances

The coefficients of the models were shown in Table 3. The assumption of linearity in the logit was met for all continuous predictors, indicating that there were no transformations necessary. The shrinkage factors were very close to 1, indicating no overfit (s = 0.99).

**Table 3:** The predictors in the different survival prediction models with the coefficients calculated with logistic regression in the development set, including the discriminative ability of each of the models in the development set and validation set and among the elderly trauma patients.

**Predictor**		**Model 1**		**Model 2**		**Model 3**		**Model 4**		**Model 5**		**Model 6**	
			coefficient		coefficient		coefficient		coefficient		coefficient		coefficient
**GCS coded^a^**		linear	0.710	linear	0.678	linear	0.788	linear	0.745	linear	0.769	linear	0.728
**SBP coded^a^**		linear	0.311	linear	0.326	linear	0.361	linear	0.351	linear	0.393	linear	0.383
**RR coded^a^**		linear	0.560	linear	0.598	linear	0.610	linear	0.620	linear	0.654	linear	0.656
**ISS**		linear	-0.111	linear	-0.122	linear	-0.127	linear	-0.134	linear	-0.127	linear	-0.133
**Age**		0–54	ref	0–54	ref	0–9	0.190	0–9	0.112	linear	-0.074	linear	-0.059
		>54	-2.788	>54	-1.779	10–19	0.073	10–19	-0.015				
						20–29	-0.173	20–29	0.007				
						30–39	ref	30–39	ref				
						40–49	1.046	40–49	1.276				
						50–59	-1.400	50–59	-0.759				
						60–69	-1.986	60–69	-1.236				
						70–79	-3.155	70–79	-2.208				
						80–89	-3.917	80–89	-2.803				
						90+	-4.379	90+	-3.222				
**ASA^b^**				ASA-1	ref			ASA-1	ref			ASA-1	ref
				ASA-2	-1.232			ASA-2	-0.810			ASA-2	-0.718
				ASA-3	-2.343			ASA-3	-1.695			ASA-3	-1.632
				ASA-4	-3.074			ASA-4	-2.585			ASA-4	-2.513
**Constant**			0.894		1.549		1.060		1.434		3.301		3.363
**R^2^**		0.27		0.32		0.33		0.35		0.32		0.35	
**AUROC (95% CI)^c^**													
	Development	0.85 (0.84, 0.87)		0.89 (0.88, 0.90)		0.89 (0.88, 0.90)		0.91 (0.89, 0.92)		0.89 (0.88, 0.90)		0.91 (0.89, 0.92)	
	Validation	0.85 (0.83, 0.87)		0.90 (0.89, 0.92)		0.88 (0.87, 0.90)		0.91 (0.90, 0.92)		0.88 (0.87, 0.90)		0.91 (0.90, 0.93)	
**AUROC (95% CI)–the elderly^d^**													
	Validation	0.68 (0.65, 0.72)		0.78 (0.75, 0.81)		0.70 (0.66, 0.74)		0.78 (0.75, 0.81)		0.70 (0.66, 0.74)		0.78 (0.75, 0.81)	
**AUROC (95% CI)–with hip#^e^**													
	Validation	0.52 (0.46, 0.58)		0.71 (0.66, 0.76)		0.62 (0.56, 0.67)		0.73 (0.69, 0.78)		0.62 (0.57, 0.68)		0.74 (0.69, 0.78)	

Abbreviations: ASA, Anesthesiologists Physical Status; AUROC, Area Under Receiver Operating Characteristic; CI, Confidence Interval; GCS, Glasgow Coma Scale; ISS, Injury Severity Score; ref, reference group; R^2^, Nagelkerke R-square; RR, Respiratory Rate; SBP, Systolic Blood Pressure.

^a^Variables were coded according to the Revised Trauma Score calculations.

^b^ASA classification; ASA-1: a normal healthy patient, ASA-2: a patient with mild systemic disease, ASA-3: a patient with severe systemic disease, ASA-4: a patient with severe systemic disease that is a constant threat to life.

^c^AUROC (95% CI) in the total development set (n = 15,530) and validation set (n = 15,504).

^d^AUROC (95% CI) in the elderly of the validation set (n = 5,405).

^e^AUROC (95% CI) in the hip fracture cohort of the validation set (n = 2,696). Patients with hip fractures were defined as ≥65 years with AIS08-codes 853161.3, 853162.3, 853151.3 and 853152.3, and ISS≤13.

The explained variance in model 1 was lower compared to all other models (R^2^: 0.27 vs. 0.32 to 0.35 respectively) (Table 3). The highest R square was found in model 4 (R^2^: 0.35).

The discriminative ability of the models for the total validation dataset and its subsets were shown in Table 3. Discrimination improved significantly after restructuring the age component (from AUROC 0.85 [95% CI: 0.83, 0.87] for model 1 to 0.88 [95% CI: 0.87, 0.90] for model 5 with age as linear predictor) (Table 3). After inclusion of the ASA classification, the discriminative ability increased to 0.91 (95% CI: 0.90, 0.93). The validation subset with the elderly showed an discriminative ability of 0.68 (95% CI: 0.65, 0.72) for model 1, with an significant increase of discriminative ability for model 6 (0.78 [95% CI: 0.75, 0.81]). The validation hip fracture cohort showed a significant increase in discriminative ability between model 1 and model 6 (AUROC: 0.52 [95% CI: 0.46, 0.58] and AUROC: 0.74 [95% CI: 0.69, 0.78] respectively). The inclusion of ASA in the prediction models showed significant higher discriminative abilities in the two subsets.

**Fig 1:**
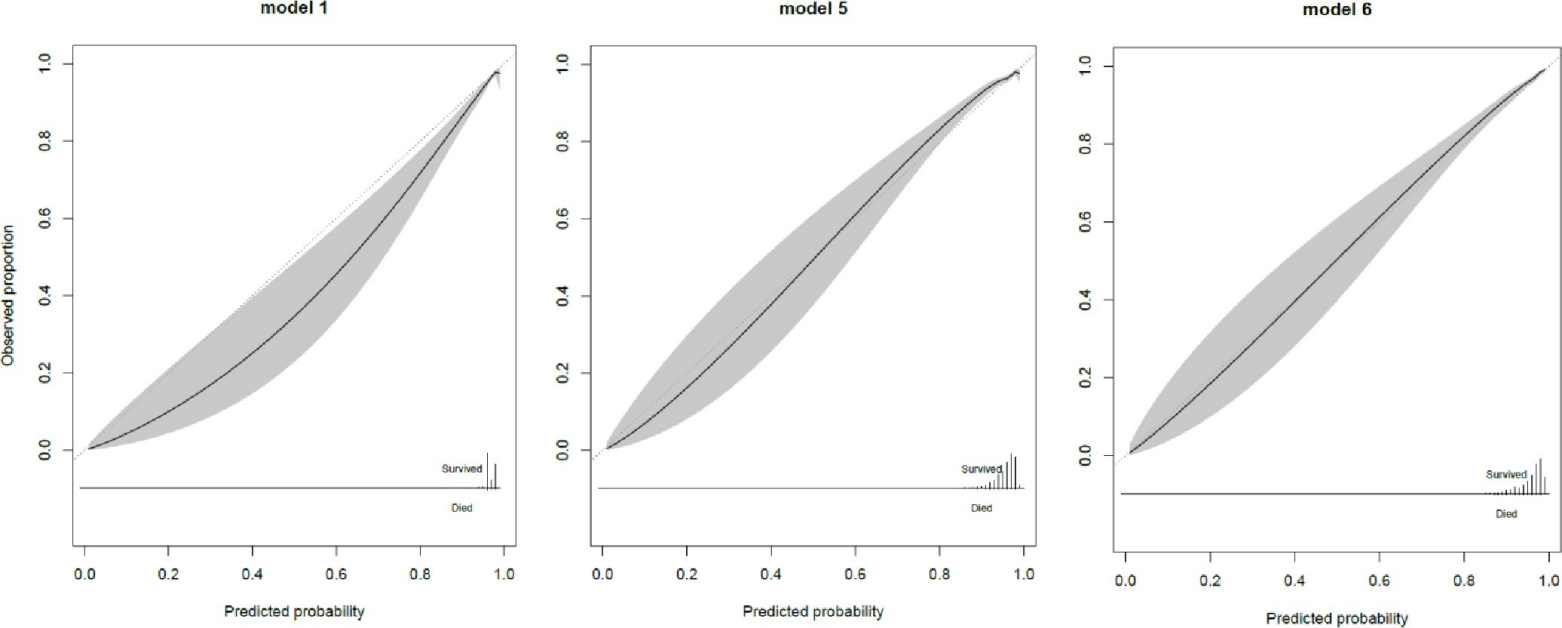
Calibration curves of model 1 (left), model 5 (middle) and model 6 (right) in the elderly subset (≥ 75 years) of the validation cohort.

Calibration curves for the elderly in the validation set were shown in Fig 1. There was an overestimation of the survivors in the elderly for model 1. The models that incorporate age as categorical or continuous predictor improved calibration. No differences were found between the calibration curves with categorical or continuous age predictor (results not shown). Including ASA as predictor in addition to the age variable showed a small improvement in calibration.

### Final model

The final model was developed in a combination dataset (n = 31,034) including both the development as the validation set, because baseline characteristics and model performances were equal in both datasets (Tables 2 and 3). Year of injury was not significant as predictor with a coefficient close to 0, and was therefore excluded from the model. ASA and age (continuous) were included in the final model, based on the best performances from the validation study. The shrinkage factor indicated no overfit (s = 1.00). The formula and coefficients of the final model are presented below:

$$P(survival)=\frac{1}{1+e^{-b}}$$

With b = 4.418 + 0.747*GCS + 0.273*SBP + 0.411*RR– 0.133*ISS– 0.055*Age– 0.546*ASA 2–1.626*ASA 3–2.929*ASA 4

**Fig 2:**
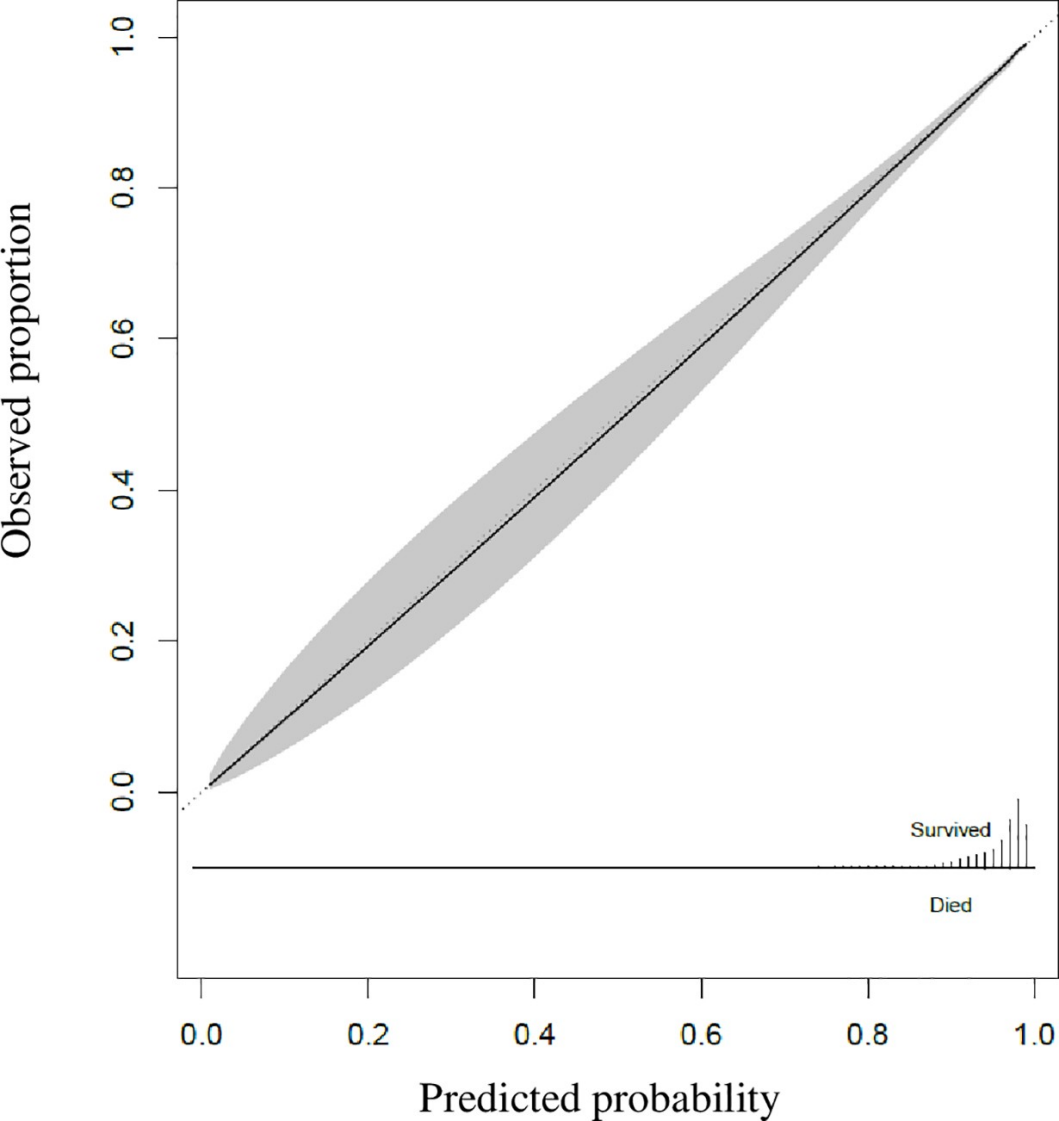
Calibration curve of the final model in the elderly subset (≥ 75 years) of the validation cohort.

R square for the final model was 0.35 with a AUROC of 0.91 (95% CI: 0.90, 0.92). The AUROC was 0.78 (95% CI: 0.76, 0.80, n = 10,774) in the elderly subset and 0.73 (95% CI: 0.70, 0.76, n = 5,288) for elderly patients (≥ 65 years) with hip fracture. The calibration curve showed high agreement between observed survival proportions and predicted survival probabilities in the elderly (Fig 2).

## Discussion

Adequate predictions are necessary to compare the quality of care between centers. It has been shown previously that the updated TRISS is not an adequate prediction model in the elderly trauma population. To provide more accurate predictions in trauma subsets in the current ageing trauma population, we believe that only small adjustments in the TRISS methodology could be sufficient, without developing a complex new model. This study showed that small adjustments of the traditional TRISS model improved the predictive performance, especially in the elderly.

Many different models were developed to provide accurate predictions for trauma populations around the world[29]. Although TRISS has several known shortcomings, it is still one of the international standards for evaluating the quality of trauma care and showed to be adequate for survival prediction in general[29–31]. Survival predictions of the updated TRISS in different subsets of the trauma population showed overestimation of survival in the older trauma patients. This implies that the quality of care in hospitals that mainly treat elderly patients seems to be worse than hospitals treating younger patients. These misleading outcomes could be adjusted by incorporating simple available variables in the formula, i.e. age as categorical (with more than 2 categories) or as a continuous variable in the TRISS. Although some studies showed an equivalent performance after these adjustments of age in the TRISS model[14,32], others showed better predictive ability[33,34]. The latter is also reflected in this study. The models showed an improvement of predictive ability in the general trauma population and calibration of the adjusted models improved significantly in the elderly. For benchmark purposes, re-categorization or restructuring of age is a beneficial small adjustment to improve survival predictions and benchmark numbers.

In addition, the elderly trauma population suffers often from comorbidities. Comorbidity can be expressed in many different ways. Prediction models that incorporate comorbidity include for example ASA and the Charlson Comorbidity Index[18,35–38]. Comorbidity can also be dichotomized or incorporated as a continuous variable; in which the presence of comorbidity or the amount of comorbidities are measured respectively[14–16,39,40]. Data on comorbidity in trauma patients has to be collected manually and is an extensive and time consuming effort. ASA classification is automatically coded in the medical records of patients who needed surgery and could relatively easy be included in the trauma registry. However, previous research showed some contradictions concerning ASA. On the one hand, the ASA scale is suggested to be a reliable mean of classifying pre-existing comorbidity in trauma patients[40] and showed to be an independent predictor of mortality after trauma[39]. On the other hand, it is suggested that ASA is a subjective and inconsistent measure, which could vary between observers[41–43]. It is therefore possible that other comorbidity measures provide different results compared to ASA. Nevertheless, this study showed an improvement of the predictive ability after including ASA in the prediction models, especially in the elderly subset with a hip fracture.

This retrospective study has several limitations. Although the discriminative ability of the new model in elderly patients with hip fracture was adequate (AUROC of 0.73), it could be much higher. Other variables are considered important predictors for mortality in geriatric trauma patients (e.g. frailty and AMTS) [10,44,45]. The Dutch Trauma Registry did not incorporate these measures, hence comparison between other models and this new presented model could not be made. However, this model is used as prognostic tool for the evaluation of trauma care, based on a population wide registry and is not used for diagnostic purposes. Therefore, we believe the high agreement between observed survival and predicted survival probabilities as shown in the calibration curves is of more importance. In addition, this study used in-hospital mortality as outcome measure. This outcome could be subject to bias by differences in hospital discharge practices[46]. Hospitals in which patients were longer admitted might have higher in-hospital mortality rates compared to hospitals in which patients were quickly discharged to other facilities. However, the alternative, e.g. 30-day mortality, is only incorporated in the Dutch trauma registry from 2014 onwards and is often missing (40% in 2014 and 24% in 2015).

## Conclusion

The inclusion of age as categorical or continuous predictor and ASA in survival prediction is a simple and effortless adjustment of the TRISS methodology to improve predictive ability and calibration in the ageing Dutch blunt trauma population. A new model is presented, through which even patients with isolated hip fractures could be included in the evaluation of trauma care.

## Abbreviations

AIS: Abbreviated Injury Scale
AMTS: abbreviated mental test score
ASA: Anesthesiologists Physical Status
AUROC: Area Under The Receiver Operating Characteristic
CI: Confidence Interval
E: Eye component of the Glasgow Coma Scale
ED: Emergency department
GCS: Glasgow Coma Scale
IQR: Inter Quartile Range
ISS: Injury Severity Score
M: Motor component of the Glasgow Coma Scale
RR: Respiratory Rate
SD: Standard deviation
SBP: Systolic Blood Pressure
TRISS: Trauma and Injury Severity Score
V: Verbal component of the Glasgow Coma Scale
